# Editorial: Special Issue “Protein Modeling and Simulation: Selected Articles from the Computational Structural Bioinformatics Workshop 2021”

**DOI:** 10.3390/biom13030408

**Published:** 2023-02-22

**Authors:** Negin Forouzesh, Kamal Al Nasr

**Affiliations:** 1 Department of Computer Science, California State University, Los Angeles, CA 90032, USA; 2 Department of Computer Science, Tennessee State University, Nashville, TN 37209, USA

Computational structural biology has demonstrated a key role in improving human health. By harnessing big data techniques and machine learning methods, computational structural biology has shed light on molecular interactions and mechanisms. The main goal of the *Biomolecules* Special Issue on Protein Modeling and Simulation was to encourage the authors of selected articles from the Computational Structural Bioinformatics Workshop (CSBW) 2021 to publish the extended version of their manuscripts. Eight articles were published in this Special Issue with topics relevant to protein structure, protein–ligand interactions, protein mutations, and molecular dynamics simulation.

With the release of AlphaFold2, we can now determine a protein tertiary structure with a high accuracy. Despite protein molecules’ high structural plasticity, a single-structure view is insufficient. In computational structural biology, obtaining a multi-structure view of a protein molecule is still challenging. In [1], Alam and Shehu advance the capability of deep learning-based models to learn from the experimentally available tertiary structures of protein molecules of varying lengths. The authors propose their beta-CVAE-SPP, a variational autoencoder neural network model, where they disentangle the learned latent factors and analyze in detail the role of the size, quality, and composition of the training dataset on the neural network’s ability to learn key local and distal patterns in protein tertiary structures. Their findings have significant implications for generative deep learning research and the ability of generative models to compute realistic multi-structure views of protein molecules.

As it was estimated that about 30% of the FDA-approved drugs in the United States target G protein-coupled receptors (GPCRs), understanding the interactions between GPCRs and their ligands is important for providing a theoretical basis for new drug design and discovery. In [2], Dankwah et al. developed a computational workflow to examine GPCRs in different families to look for characteristics that allow these proteins to bind to the same ligand. Their analysis showed that the same ligand can bind to GPCR molecules with diverse amino acid sequences and different overall structures, but the binding pockets of these GPCRs are locally similar in their electrostatic as well as their three-dimensional structural properties.

Interactions between proteins and small molecules, called ligands, govern vital biological processes in the human body. It also plays a key role in molecular recognition that is central to drug design and discovery. One key factor for studying protein–ligand interactions is the strength of binding, or affinity, which could be measured as the binding free energy. Classic implicit solvent models, which have been widely used to accomplish this task, lack accuracy compared to experimental references. Emerging data-driven models, on the other hand, are often accurate, yet not fully interpretable, and are likely to be overfitted. In [3], Cain et al. explored the application of theory-guided data science in studying protein–ligand binding. A hybrid model is introduced by integrating a graph convolutional network (data-driven model) with the GBNSR6 implicit solvent (physics-based model). The results demonstrate that the proposed model can improve the “accuracy” of the pure physics-based model. In addition, the “interpretability” and “transferability” of this model have been boosted compared to the purely data-driven model.

The advancement of AlphaFold2 to computational models, which can now be as accurate as experimentally resolved models, signals the emergence of several next holy grails in computational structural biology. An essential one in this direction is to move from a single-structure to a multi-structure view of protein molecules as obtaining a detailed understanding of the structure and dynamics of unbound and bound molecular systems remains a fundamental challenge. In [4], Kabir et al. investigated the utility of a coarse-grained representation (based on ultrafast shape recognition) as a better state space discretization to obtain Markov State models of a higher quality with the capability to capture the multi-view structural dynamics of biological molecules and their complex, even in the absence of the associated energetics.

In [5], Jilani et al. report on computational studies on the effects of amino acid insertion and deletion (InDels) mutations. They employ inverse kinematics, ab initio energy minimization, and rigidity analysis to generate InDels and analyze their effects. In their work, they also study how structural effects due to InDels differ from the effects of amino acid substitutions, which is another form of protein mutation. Lastly, they perform a correlation analysis between a rigidity-based metrics approach and wet lab data for their ability to infer the effects of InDels on protein fitness.

To characterize the structural arrangement of cytoskeletal filaments in 3D maps of subcellular components, Sazzed et al. presented a framework named Spaghetti Tracer [6], which uses a dynamic programming (DP)-based algorithm. Spaghetti Tracer incorporates the spatial domain technique that operates directly on the voxels of the 3D tomogram. Spaghetti Tracer assumes that the tomogram can be rotated so that the filaments have a dominant orientation, and then uses this assumption to identify locally high-density seed points as the starting points for potential filament segments. Afterward, by incorporating a DP-based bipyramidal density accumulation algorithm, short filament segments are generated. The segment selection step then identifies likely true filament segments that are fused in the merging step to create the final filament traces. The Spaghetti Tracer is tested on simulated tomograms that mimic the noise and appearance of experimental maps. Utilizing simulated maps based on a known ground truth allows for the quantitative validation of a Spaghetti Tracer. It exhibits a high efficacy in tracing actin filaments by achieving F1 scores of 0.86–0.95 under various experimental noise conditions.

Cryo-electron microscopy (cryo-EM) is a structural technique that has played a significant role in protein structure determination in recent years. Cryo-EM has some advantages over conventional methods such as X-ray crystallography and nuclear magnetic resonance (NMR). One of the advantages is the ability to visualize large protein complexes. Cryo-EM 3D images are produced at different ranges of the resolution, near-atomic, or high, medium, and low. At a medium resolution, the atomic information of protein molecules is hard to determine. Usually, computational methods are used to help in the determination of the protein structure. This set of methods is called de novo. In this technique, one intermediate step is to determine the correct topology of the protein sequence to the 3D image; in particular, determining the assignment and direction of each secondary structure element (SSE) of the sequence to some detected sticks and plates that represent the location of these elements on the cryo-EM 3D image. In [7], Behkamal et al. proposed a novel automatic computational method to address the topology determination problem. Initially, they did this through modeling the target sequence with the aid of extracting highly reliable features from a generated 3D model and the image. The SSEs matching problem is formulated as a 3D vector matching problem. Afterward, the 3D vector matching problem is transformed into a 3D graph matching problem. Finally, a similarity-based voting algorithm combined with the principle of least conflict (PLC) concept is developed to obtain the SSEs correspondence. Comparative studies are conducted to demonstrate the superiority of the proposed method over some state-of-the-art techniques.

In [8], Sun investigated the genomic similarity of SARS-CoV-2 to humans and identifies genetic mutations that may help the virus adapt to the host. A quantitative definition of host–genome similarity (HGS) is proposed. The HGS analysis is applied to 40237 SARS-CoV-2 genomes. The study finds that (1) a correlation exists between the HGS and the inhibition of antivirus productions. The open reading frame (ORF) with a higher HGS suppressed the gene expression of ISRE-regulated genes to a greater extent. (2) The ORF 7b and ORF 8 of SARS-CoV-2 have an apparent HGS increment during the last two years. Such HGS changes suggest that these ORFs may be important in helping SARS-CoV-2 evolve the efficient evasion of the host’s innate immune system. (3) The Delta variant has a significant HGS increment. The Omicron variant has a relatively low HGS, which is still poorly studied. The results imply that the growth of HGS in the SARS-CoV-2 genome may indicate the stronger inhibition of IFN I synthesis and delayed host innate immunity. SARS-CoV-2 has found a way to improve the host adaptation via nucleotide variations in ORFs, leading to higher HGS. HGS analysis provides a way to identify SARS-CoV-2 genes and mutations well adapted to humans, which may help in the search for potential targets for the pharmaceutical agent. The analysis indicates that HGS is a reliable indicator of the suppression of innate immunity by viral proteins. Therefore, by observing the HGS changes in the viral genome, it is possible to provide an early warning when the virus becomes more dangerous to humans. This is the first time that virus–host genome similarity has been linked to a virus’s ability to suppress the immune system.
